# Assessment of cattle genetic introgression into domestic yak populations using mitochondrial and microsatellite DNA markers

**DOI:** 10.1111/j.1365-2052.2009.01989.x

**Published:** 2010-06

**Authors:** X B Qi, H Jianlin, G Wang, J E O Rege, O Hanotte

**Affiliations:** * International Livestock Research Institute (ILRI)P.O. Box 30709, Nairobi 00100, Kenya; † State Key Laboratory of Genetic Resources and Evolution, Kunming Institute of Zoology, Chinese Academy of ScienceKunming 650223, China; ‡ CAAS-ILRI Joint Laboratory on Livestock and Forage Genetic Resources, Institute of Animal Sciences, Chinese Academy of Agricultural Sciences (CAAS)Beijing 100193, China; § Key Laboratory of Arid and Grassland Agro-Ecology, Lanzhou UniversityLanzhou 730000, China; ¶ School of Biology, University of Nottingham, University ParkNG2 2RD Nottingham, UK

**Keywords:** admixture analysis, cattle, introgression, Qinghai-Tibetan Plateau, yak

## Abstract

Hybridization between yak *Poephagus grunniens* and taurine *Bos taurus* or indicine *B. indicus* cattle has been widely practiced throughout the yak geographical range, and gene flow is expected to have occurred between these species. To assess the impact of cattle admixture on domestic yak, we examined 1076 domestic yak from 29 populations collected in China, Bhutan, Nepal, India, Pakistan, Kyrgyzstan, Mongolia and Russia using mitochondrial DNA and 17 autosomal microsatellite loci. A cattle diagnostic marker-based analysis reveals cattle-specific mtDNA and/or autosomal microsatellite allele introgression in 127 yak individuals from 22 populations. The mean level of cattle admixture across the populations, calculated using allelic information at 17 autosomal microsatellite loci, remains relatively low (*mY*_cattle_ = 2.66 ± 0.53% and *Q*_cattle_ = 0.69 ± 2.58%), although it varies a lot across populations as well as among individuals within population. Although the level of cattle admixture shows a clear geographical structure, with higher levels of admixture in the Qinghai-Tibetan Plateau and Mongolian and Russian regions, and lower levels in the Himalayan and Pamir Plateau region, our results indicate that the level of cattle admixture is not significantly correlated with the altitude across geographical regions as well as within geographical region. Although yak-cattle hybridization is primarily driven to produce F_1_ hybrids, our results show that the subsequent gene flow between yak and cattle took place and has affected contemporary genetic make-up of domestic yak. To protect yak genetic integrity, hybridization between yak and cattle should be tightly controlled.

## Introduction

The yak *Poephagus grunniens* is a member of family *Bovidae*. It is endemic to the Central Asian Highlands centred round the Qinghai-Tibetan Plateau, a vast mountainous region characterized by cold and high altitude environments (typically above 3500 metres). With a current total population size of 14 million, the domestic yak constitutes one of the most important livestock genetic resources and plays an indispensable role in the life of pastoralists and agro-pastoralists in the region (Zhang 1989; Wiener *et al.* 2003). Today, domestic yak is distributed in Central Asia extending from the southern slopes of the Himalayas to the Altai and Hangai mountains of Mongolia and Russia, and from the Pamir Plateau and Tian-shan mountains in the west to the Qi-lian and Min-shan mountains in the east.

The hybridization of yak with cattle has been documented in ancient historical records. In China, the earliest practice of hybridization between yak and local cattle is thought to have started during the Yin Dynasty (approximately 1100 B.C.) (Cai 1989; Zhang 2000; and references therein). Such hybridization is still widely practiced today in pastoral and agro-pastoral areas across the entire geographical distribution range of the species, with observations that yak-cattle F_1_ hybrid animals are superior to both parental types in many aspects. For example, the F_1_ hybrids are reported to have better beef conformation and greater size, and to produce higher milk yields as well as to have better ability to withstand a warmer climate at lower altitudes than yak (Phillips *et al.* 1946a,b; White *et al.* 1946; Joshi 1982; Zhang 2000; Wiener *et al.* 2003). Traditionally, local cattle bulls are used to interbreed naturally with yak cows at higher altitudes, while reciprocal interbreeding is more common at lower altitudes. Some European cattle breeds, such as Angus, Holstein and Simmental, among others, have also been used for the exercise since the 1940s in limited areas, and this practice has been promoted through artificial insemination using frozen-thawed semen of exotic breeds since the 1970s (The Editing-Committee 1989). Whether taurine *B. taurus* or indicine *B. indicus* cattle were involved in the hybridization largely depends on the geographical area, e.g. taurine cattle were used in the Qinghai-Tibetan Plateau and Mongolian Plateau (Phillips *et al.* 1946a,b; Cai 1989; Zhang 1989) and indicine cattle used in the Himalayan areas and elsewhere (Phillips *et al.* 1946b; Joshi 1982; Wiener *et al.* 2003).

F_1_ hybrid males are sterile, while females remain fertile. Typically, after four generations of backcrossing of hybrid cows to parental bulls, hybrid males resume their fertility and the offspring are indistinguishable from ‘pure yak’ or ‘pure cattle’ in body conformation and appearance. Therefore, some animals which resemble yak probably carry genes that have been introgressed from cattle several generations earlier (Phillips *et al.* 1946a, b).

In yak, a mitochondrial DNA (mtDNA)-specific fragment has been described (Ward *et al.* 1999), and cattle autosomal microsatellite loci are now commonly used for the study of their genetic diversity (Ritz *et al.* 2000; Dorji *et al.* 2002; Xuebin *et al.* 2002, 2005; Qi 2004; Nguyen *et al.* 2005). Recently, a mtDNA study identified taurine cattle mtDNA haplotypes in two yak samples from Tibetan and Maiwa yak populations (Lai *et al.* 2007). However, no study has reported so far the use of genetic markers to assess the occurrence, frequency and importance of cattle introgression in individual yak or in domestic yak populations across the geographical range of the species. We report here the results of cattle admixture in domestic yak populations across the entire geographical distribution range of the species using cattle-specific mtDNA haplotypes and allelic information at 17 autosomal microsatellite loci.

## Materials and methods

### Sample collection and DNA extraction

**Figure 1:**
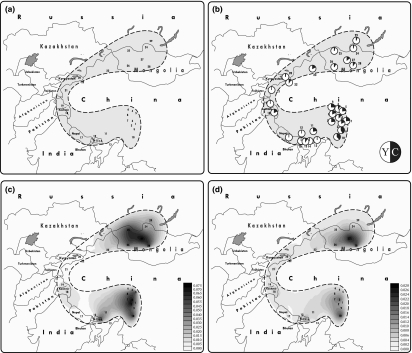
A map showing the domestic yak distributions (shaded area) and cattle introgression in domestic yak populations. a, Sampling locations: 1, Luqu; 2, Maqu; 3, Xiahe; 4, Tianzhu Black; 5, Tianzhu White; 6, Sunan; 7, Jianzha; 8, Datong; 9, Maiwa; 10, Jiulong; 11, Jiali; 12, Pali; 13, Northeast Indian; 14, East Bhutanese; 15, Central Bhutanese; 16, West Bhutanese; 17, Nepalese; 18, Northwest Indian; 19, Pakistani; 20, Kyrgyzstan; 21, Kashi; 22, Aksu; 23, Bazhou; 24, Hovsgol; 25, Ubs; 26, Gobi Altai; 27, North Hangai; 28, South Gobi; and 29, Buryatia. b, Frequency of yak individuals carrying cattle mtDNA sequences or cattle diagnostic alleles at *ILSTS013*, *ILSTS050* and *SPS115*. c, A synthetic contour map showing the cattle admixture proportion (*mY*_cattle_) in domestic yak populations. d, A synthetic contour map showing the mean cattle admixture co-efficient (*Q*_cattle_) in domestic yak populations.

A total of 1076 yak samples were collected from 29 yak populations in China, Bhutan, India, Nepal, Pakistan, Kyrgyzstan, Mongolia and Russia (Table 1 and Fig. 1a). Only phenotypically pure animals with no recent history of hybridization with cattle, as per the herder’s information, were sampled. We divided these yak populations into three major geographical groups: Qinghai-Tibetan Plateau (QTP), Himalaya and Pamir Plateau (HPP), and Mongolia and Russia (M&R), according to our previous phylogeographic analysis (Qi 2004). We further divided the QTP group into subgroups of heartland QTP and surrounding QTP according to sampling locations of yak populations either in the heartland or the surrounding areas of the Qinghai-Tibetan Plateau.

**Table 1:** The frequency of cattle mtDNA sequences and cattle-specific microsatellite alleles (%) in domestic yak populations.

Country/ area	Population	N	mtDNA	*ILSTS013*	*ILSTS050*	*SPS115*	Three microsatellite loci together^1^	MtDNA + three microsatellite loci together^1^
China	Luqu	30	3.33 (1)	0	0	20.00 (6)	20.00 (6)	23.3 (7)
	Maqu	45	0	2.22 (1)	2.22 (1)	13.33 (6)	17.78 (8)	17.8 (8)
	Xiahe	17	0	0	0	11.76 (2)	11.76 (2)	11.8 (2)
	Jianzha	34	0	2.94 (1)	0	23.53 (8)	23.53 (8)	23.5 (8)
	Datong	38	5.26 (2)	2.63 (1)	5.26 (2)	7.89 (3)	15.79 (6)	18.4 (7)
	Jiali	50	0	2.00 (1)	0	22.00 (11)	22.00 (11)	22.0 (11)
	Bazhou	51	0	0	0	7.84 (4)	7.84 (4)	7.8 (4)
Heartland QTP		265	1.13 (3)	1.51 (4)	1.13 (3)	15.09 (40)	16.98 (45)	17.78 (47)
China	Tianzhu Black	46	10.87 (5)	4.35 (2)	2.17 (1)	4.35 (2)	10.87 (5)	19.6 (9)
	Tianzhu White	48	2.08 (1)	0	6.25 (3)	14.58 (7)	20.83 (10)	22.9 (11)
	Sunan	36	5.56 (2)	5.56 (2)	0	13.89 (5)	19.44 (7)	25.0 (9)
	Maiwa	24	8.33 (2)^2^	16.67 (4)	0	16.67 (4)	33.33 (8)	41.7 (10)
	Jiulong	24	4.17 (1)	29.17 (7)	0	4.17 (1)	33.33 (8)	37.5 (9)
Surrounding QTP		178	6.18 (11)	8.43 (15)	2.25 (4)	10.67 (19)	21.35 (38)	26.97 (48)
Qinghai-Tibet Plateau (QTP) overall		443	3.16 (14)	4.29 (19)	1.58 (7)	13.32 (59)	18.74 (83)	21.44 (95)
China	Pali	46	0	0	0	0	0	0
	Kashi	47	0	0	0	0	0	0
	Aksu	31	0	6.45 (2)	3.23 (1)	0	9.68 (3)	9.7 (3)
India	Northeast Indian	21	0	0	0	0	0	0
	Northwest Indian	44	0	15.91 (7)	0	2.27 (1)	18.18 (8)	18.2 (8)
Bhutan	East Bhutanese	32	0	0	0	0	0	0
	Central Bhutanese	32	3.13 (1)	3.13 (1)	0	3.13 (1)	6.25 (2)	9.4 (3)
	West Bhutanese	33	0	0	0	0	0	0
Nepal	Nepalese	25	0	–	–	–	–	–
Pakistan	Pakistani	50	0	0	0	0	0	0
Kyrgyzstan	Kyrgyzstan	44	0	0	0	2.27 (1)	2.27 (1)	2.3 (1)
Himalaya and Pamir Plateau (HPP)		405	0.25 (1)	2.63(10)	0.25 (1)	0.79 (3)	3.46 (14)	3.70 (15)
Mongolia	Hovsgol	40	0	2.50 (1)	0	0	2.50 (1)	2.5 (1)
	Ubs	30	0	0	3.33 (1)	0	3.33 (1)	3.3 (1)
	Gobi Altai	38	2.63 (1)	5.26 (2)	0	0	5.26 (2)	7.9 (3)
	North Hangai	49	4.08 (2)	6.12 (3)	6.12 (3)	2.04 (1)	10.20 (5)	14.3 (7)
	South Gobi	31	0	6.45 (2)	0	3.23 (1)	9.68 (3)	9.7 (3)
Russia	Buryatia	40	2.50 (1)	2.50 (1)	0	0	2.50 (1)	5.0 (2)
Mongolia and Russia (M&R)		228	1.75 (4)	3.95 (9)	1.75 (4)	0.88 (2)	5.70 (13)	7.46 (17)
Grand total		1076	1.77 (19)	3.62 (38)	1.14 (12)	6.09 (64)	10.22 (110)	11.80 (127)

The numbers of yak individuals showing cattle introgression are given in the parentheses. N, sample size; -, no PCR amplification.

1 Individual animal showing more than one cattle-specific mtDNA or autosomal microsatellite alleles at *ILSTS013*, *ILSTS050* and *SPS115* is counted as one individual when calculating the individual frequency of cattle introgression.

2 No mtDNA control region sequence obtained.

Genomic DNA was extracted following the methods described in Sambrook *et al.* (1989) for blood samples, Xuebin *et al.* (2005) for blood on Whatman FTA cards (Whatman BioScience) and Troy *et al.* (2001) for hair root samples. In addition, two Chinese local taurine cattle populations (Tibetan cattle, *n* = 26 and Wuwei cattle, *n* = 40) and one yak-cattle F_1_ hybrid population (*n* = 41) were also included as reference populations.

### MtDNA control region amplification and sequencing

Cattle mtDNA in yak was detected through the amplification of a 357-bp taurine and indicine cattle-specific mtDNA control region fragments using primers MTD1 (5′-AGCTAACATAACACGCCCATAC-3′) and MTD2 (5′-CCTGAAGAAAGAACCAGATGC-3′) (Ward *et al.* 1999) in a multiplex PCR reaction also containing primers MTR1 (5′-CCCGCCTGTTTATCAAAAACAT-3′) and MTR2 (5′-CCCTCCGGTTTGAACTCAGAT-3′) (Derr *et al.* 1992), which amplified a 590-bp mammalian-conserved *16S* rDNA fragment as an internal control. The partial mtDNA control region fragment was further sequenced for cattle-specific mtDNA detected in yak populations to verify their taurine or indicine identities. The sequences of haplotypes have been deposited in the GenBank with accession numbers AY428633–AY428639 and AY428641–AY428643. PCR amplification and sequencing were carried out as described in Appendix S1.

### Microsatellite loci genotyping

Seventeen unlinked bovine microsatellite loci, selected from the BovMAP database, INRA, France (http://locus.jouy.inra.fr/cgi-bin/bovmap/intro.pl) were used to genotype all the samples as described in Xuebin *et al.* (2005), with the annealing temperatures given in Table S1. These microsatellite loci and their allele size ranges in domestic yak are given in Table S1 and raw data are available from the corresponding author. The Nepalese yak population, consisting of hair samples only, was excluded from this analysis because of their poor amplification at the majority of microsatellite loci. Of these microsatellite loci, three loci (*ILSTS013*, *ILSTS050* and *SPS115* localized on bovine chromosomes 9, 2 and 15, respectively) yielded complete distinct allelic patterns between yak and cattle (Fig. S1), and they were therefore used as diagnostic markers for detecting cattle introgression in yak. The allele size differences at these loci between yak and cattle were further confirmed by sequencing of selected alleles.

### Cattle admixture analysis

Three methods were used to assess the impact of cattle introgression on domestic yak populations. (i) A cattle diagnostic marker-based method, using cattle mtDNA sequences and cattle-specific alleles at microsatellite loci of *ILSTS013*, *ILSTS050* and *SPS115*, was used to assess the frequency of occurrence of cattle introgression in domestic yak populations at the population level. (ii) The level of cattle admixture in yak populations was assessed using an admixture estimator, *mY*, which is the relative contribution of two parental populations to a hybrid population and estimated using frequencies and size information of microsatellite alleles. It was initially described in Bertorelle & Excoffier (1998) and extended to any number of parental populations by Dupanloup & Bertorelle (2001). It was calculated using the program admix 2.0 (Dupanloup & Bertorelle 2001) with the two Chinese taurine cattle populations (*n* = 66) and the six yak populations (Pali, Kashi, Northeast Indian, East Bhutanese, West Bhutanese and Pakistani) located in the Himalaya and Pamir Plateau, showing no presence of cattle-specific mtDNA and alleles at *ILSTS013*, *ILSTS050* and *SPS115* loci (Table 1), used as parental populations for this analysis. (iii) In addition, a model-based Bayesian clustering algorithm that employs a Markov Chain Monte Carlo (MCMC) method to estimate the posterior distribution (*Q*) of each individual’s admixture co-efficient was used. It allows us to assess the level of cattle introgression at population as well as at individual levels. The estimator *Q* represents an estimate of the amount of an individual’s genome that is derived from one of the inferred parental populations (Pritchard *et al.* 2000). This analysis required no prior information to characterize the parental populations or to assign individuals to those populations, and was performed using allelic information at 17 autosomal microsatellite loci. It was inferred with the program structure 2.1 (Pritchard *et al.* 2000) with parameters *K*=2, a burn-in of 500 000 repetitions, and run length of 1 000 000. A Mann–Whitney *U*-test was applied to examine the difference of cattle introgression level among different geographical groups of yak.

## Results

### Cattle mtDNA analysis

A total of 19 cattle mtDNA sequences (1.8%) were detected in 1076 yak. More particularly, cattle mtDNA was detected in 11 out of the 29 yak populations with a within-population frequency ranging from 2.1% (*n* = 1, Tianzhu White) to 10.9% (*n* = 5, Tianzhu Black). Of the 19 yak carrying cattle mtDNA, 11 were males and eight females. These cattle mtDNA sequences were predominantly observed in the QTP (*n* = 14 or 3.16% of the animals) and M&R (*n* = 4 or 1.75% of the animals) groups, and only sparsely in the HPP group (*n* = 1 or 0.25% of the animals). The QTP group had a significantly higher frequency of cattle mtDNA sequences than the HPP group (*P*<0.05), however, the frequency was not statistically different between the QTP and M&R (*P*=0.10) groups and between the M&R and HPP (*P*=0.35) groups. The frequency of yak with cattle mtDNA sequences in the surrounding QTP subgroup (6.18%) was significantly higher than that in the heartland QTP subgroup (1.13%) (*P*<0.05) (Table 1).

To further verify the taurine or indicine origin of cattle mtDNA introgression in yak, a partial mtDNA control region sequence (486 bp) was obtained for 17 out of the 19 cattle mtDNA genomes detected in the yak populations. We failed to obtain mtDNA sequences for two samples from the Chinese Maiwa yak population. Comparison of the 17 cattle mtDNA sequences detected in yak populations with published cattle sequences (Troy *et al.* 2001) indicated their taurine cattle origin for all. The phylogenetic analysis further assigned these sequences into 10 haplotypes defined by polymorphisms at 11 sites. One haplotype (AY428637) occurred seven times (four in Tianzhu Black, two in Datong and one in Gobi Altai population), another one (AY428636) occurred twice (one in Tianzhu Black and one in Central Bhutanese population), and the remaining eight haplotypes only occurred once. According to the haplotype definitions described in Troy *et al.* (2001), these 10 haplotypes belong to the T3 haplogroup, which predominates in European cattle (Troy *et al.* 2001). Similarly, the T3 haplogroup also predominates in Chinese taurine cattle (Lai *et al.* 2006), and therefore it is not possible to determine whether these haplotypes detected in yak populations were introgressed from either European or Chinese local taurine cattle.

### Analysis of cattle-specific microsatellite alleles in domestic yak populations

As illustrated in Fig. S1, the allelic genotype patterns overlapped between yak and cattle in 14 out of the 17 microsatellite loci genotyped, therefore it was not possible to determine the cattle-specific alleles at these loci. However, three microsatellite loci (*ILSTS013*, *ILSTS050* and *SPS115*) yielded allele sizes that were completely distinct between yak and cattle (Appendix S1). These alleles have been further sequenced to confirm their size differences between yak and cattle (Feng *et al.* 2009).

A total of 26 yak-specific and 29 cattle-specific alleles were amplified at *ILSTS013*, *ILSTS050* and *SPS115* loci in yak, cattle and yak-cattle F_1_ hybrid populations. Of the 29 cattle-specific alleles, 16 were detected in 22 out of the 28 yak populations (the Nepalese yak population was excluded from admixture analyses because of their poor amplification at the majority of microsatellite loci). The frequency of these cattle-specific alleles in yak populations varies widely among loci (Table 1). More precisely, six cattle-specific alleles were detected at *ILSTS013* in 38 yak individuals from 16 populations, and the frequency of yak carrying cattle alleles varied from 2.0% (*n* = 1, Chinese Jiali) to 29.2% (*n* = 7, Chinese Jiulong). It was not statistically different among the QTP, HPP and M&R groups (*P*>0.05). At *ILSTS050*, five cattle-specific alleles were detected in a total of 12 yak individuals from seven populations with frequencies ranging from 2.2% to 6.3% and similar among the QTP, HPP and M&R groups (*P*>0.05). Five cattle-specific alleles at *SPS115* were detected in 64 yak individuals from 17 populations. The frequency was significantly higher in the QTP (13.32%) than in the HPP (0.79%) and M&R (0.88%) groups (*P*<0.001) while there was no difference between the HPP and M&R groups (*P*>0.05). As observed in introgressed cattle mtDNA sequences, this cattle-specific autosomal microsatellite allele-based method detected cattle introgression events predominantly in the QTP and M&R yak groups. However, unlike the mtDNA results, the frequency of cattle introgression detected in the heartland QTP and surrounding QTP subgroups was similar (*P*>0.05) at each of the three microsatellite loci. When the data at three microsatellite loci were combined together, the frequency of cattle introgression was significantly higher in the QTP group (18.74%) than in the HPP (3.46%) and M&R (5.70%) groups (*P*<0.01), while no difference was detected between the HPP and M&R groups (*P*=0.09), and between the heartland QTP (16.98%) and surrounding QTP (21.35%) subgroups (*P*=0.33).

### Combined analysis of cattle-specific mtDNA and autosomal microsatellite alleles

We combined the data from mtDNA and three diagnostic microsatellite loci (*ILSTS013*, *ILSTS050* and *SPS115*) to calculate the frequency of yak carrying cattle genes introgressed from mitochondrial and/or nuclear genome in each population. A total of 127 or 11.80% yak from 22 populations were found to have introgressed cattle mtDNA sequences and/or autosomal microsatellite alleles (Table 1). Some yak individuals showing cattle mtDNA sequences do present a typical yak microsatellite profile (Table S2). The distribution of the frequency of cattle-specific mtDNA sequences and microsatellite alleles is shown in Fig. 1b. The frequency at the population level ranged from 2.3% (Kyrgyzstan yak) to 41.7% (Chinese Maiwa yak). Cattle-specific sequences/alleles were absent in six HPP yak populations (Pali, Kashi, Northeast Indian, East Bhutanese, West Bhutanese and Pakistani yak). This combined analysis indicated a significantly higher frequency of cattle introgression in the QTP group (21.44%) than in the M&R (7.46%) and HPP groups (3.70%) (*P*<0.01). This frequency was also significantly higher in the surrounding QTP (26.97%) subgroup than in the heartland QTP (17.78%) subgroup (*P*<0.05). The M&R group also showed a relatively higher frequency of cattle introgression than the HPP group (*P*=0.084). Cattle introgression was found in four out of the 10 yak populations in the HPP areas (Northwest Indian, Central Bhutanese, Aksu and Kyrgyzstan) with frequencies ranging from 2.27% to 18.18% (Table 1).

### Cattle admixture analysis in domestic yak populations

**Figure 2:**
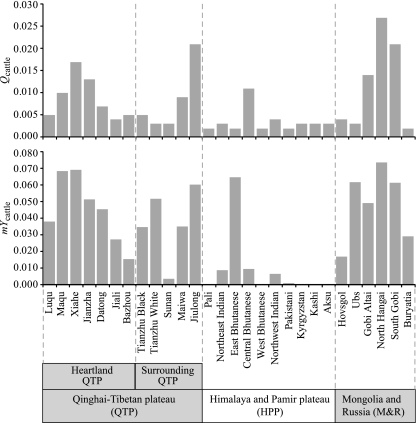
The variation of the cattle admixture in domestic yak populations using an allele frequency-based admixture analysis (*mY*_cattle_) and a model-based Bayesian clustering method (*Q*_cattle_). Note that the Bazhou yak, sampled from north part of the Xinjiang province of China, were originally introduced from the Qinghai-Tibetan Plateau (Wiener *et al.* 2003 and references therein), and therefore this population was classified into the Qinghai-Tibetan Plateau group in this study.

To assess the level of cattle introgression in domestic yak populations at genome level, we determined the mean cattle admixture proportion in domestic yak populations using admixture analyses based on both allele frequency and allele size information (*mY*), and a model-based Bayesian clustering algorithm. We obtained negative estimates of *mY* in five yak populations of Pali, Kashi, Aksu, West Bhutanese and Kyrgyzstan as well as in the HPP group, and these negative *mY* values were set to zero. The *mY* analysis detected an average proportion of 2.66 ± 0.53% cattle genetic admixture in domestic yak populations, with values ranging from zero (Pali, Kashi, Aksu, West Bhutanese and Kyrgyzstan) to 7.36% (North Hangai). Similar levels of mean cattle admixture proportions were detected in the QTP (3.90 ± 0.62%) and M&R (4.78 ± 0.75%) groups (*P*=0.45), and they were significantly higher than that in the HPP group (0.00 ± 0.58%, *P*<0.01) (Table 2, Fig. 1c). There was no difference in the level of cattle admixture between the heartland QTP and surrounding QTP subgroups (*P*>0.05). Fig. 2 shows the variation of the mean cattle admixture proportion in terms of *mY* among the 28 yak populations and geographical groups. As compared with the diagnostic marker-based method and excluding the populations with negative values of *mY*, admixture analysis also detected cattle introgression in Northeast Indian, East Bhutanese and Pakistani yak populations, in which no cattle-specific mtDNA sequence or autosomal microsatellite alleles at *ILSTS013*, *ILSTS050* and *SPS115* were detected.

**Table 2:** Cattle admixture analysis in domestic yak populations using allelic information at 17 autosomal microsatellite loci.

Country/area	Population	N	*mY*_cattle_ (SD)	*Q*_cattle_ (SD)
China	Luqu	30	0.0383 (0.0167)	0.0050 (0.0141)
	Maqu	45	0.0687 (0.0147)	0.0100 (0.0225)
	Xiahe	17	0.0691 (0.0231)	0.0171 (0.0384)
	Jianzha	34	0.0516 (0.0162)	0.0133 (0.0359)
	Datong	38	0.0455 (0.0155)	0.0074 (0.0121)
	Jiali	50	0.0275 (0.0113)	0.0035 (0.0069)
	Bazhou	51	0.0154 (0.0119)	0.0048 (0.0106)
Heartland QTP		265	0.0417 (0.0070)	0.0077 (0.0206)
	Tianzhu Black	46	0.0349 (0.0142)	0.0045 (0.0083)
	Tianzhu White	48	0.0518 (0.0147)	0.0028 (0.0023)
	Sunan	36	0.0039 (0.0135)	0.0034 (0.0034)
	Maiwa	24	0.0351 (0.0197)	0.0090 (0.0199)
	Jiulong	24	0.0603 (0.0200)	0.0213 (0.0422)
Surrounding QTP		178	0.0353 (0.0084)	0.0067 (0.0185)
Qinghai-Tibetan Plateau (QTP) overall		443	0.0390 (0.0062)	0.0073 (0.0197)
China	Pali	46	0.0000 (0.0106)	0.0024 (0.0024)
	Kashi	47	0.0000 (0.0113)	0.0031 (0.0049)
	Aksu	31	0.0000 (0.0127)	0.0030 (0.0039)
India	Northeast Indian	21	0.0087 (0.0167)	0.0025 (0.0036)
	Northwest Indian	44	0.0066 (0.0129)	0.0038 (0.0055)
Bhutan	East Bhutanese	32	0.0648 (0.0160)	0.0015 (0.0005)
	Central Bhutanese	32	0.0096 (0.0117)	0.0107 (0.0290)
	West Bhutanese	33	0.0000 (0.0111)	0.0019 (0.0009)
Nepal	Nepalese	–	–	–
Pakistan	Pakistani	50	0.0011 (0.0105)	0.0020 (0.0023)
Kyrgyzstan	Kyrgyzstan	44	0.0000 (0.0112)	0.0025 (0.0029)
Himalaya and Pamir Plateau (HPP)		380	0.0000 (0.0058)	0.0032 (0.0092)
Mongolia	Hovsgol	40	0.0171 (0.0138)	0.0042 (0.0074)
	Ubs	30	0.0619 (0.0147)	0.0035 (0.0070)
	Gobi Altai	38	0.0491 (0.0143)	0.0143 (0.0480)
	North Hangai	49	0.0736 (0.0143)	0.0268 (0.0718)
	South Gobi	31	0.0617 (0.0164)	0.0208 (0.0648)
Russia	Buryatia	40	0.0292 (0.0144)	0.0024 (0.0019)
Mongolia and Russia (M&R)		228	0.0478 (0.0075)	0.0129 (0.0462)
Grand total		1051	0.0266 (0.0053)	0.0069 (0.0258)

N, sample size; SD, standard deviation; -, no data available. A negative *mY* estimate was obtained in Pali, Kashi, Aksu, West Bhutanese and Kyrgyzstan populations as well as in the HPP group, and these negative *mY* values were set to zero.

By applying a model-based Bayesian clustering algorithm using allelic information at 17 autosomal microsatellite loci, we inferred an average proportion of 0.69 ± 2.58% cattle admixture co-efficient (*Q*_cattle_) in the yak genome, with values ranging from 0.15 (East Bhutanese) to 2.68 (North Hangai). The *Q*_cattle_ was 0.73 ± 1.97% in the QTP group, which was relatively lower than that in the M&R group (1.29 ± 4.62%) (*P*=0.92) but significantly higher than that in the HPP group (0.32 ± 0.92%) (*P*<0.01). The *Q*_cattle_ was only higher in the M&R group than in the HPP group at a marginally significant level (*P*=0.065). Similar to the *mY* analysis, the *Q*_cattle_ was not statistically different between the heartland QTP (0.77 ± 2.06%) and surrounding QTP (0.67 ± 1.85%) subgroups (*P*=0.46) (Table 2). Fig. 2 shows the variation of the inferred *Q*_cattle_ among 28 yak populations and geographical groups. This model-based Bayesian clustering method also showed that the mean level of cattle admixture was much higher in the QTP and M&R groups than that in the HPP group (Fig. 1d).

At an individual animal level, the inferred *Q*_cattle_ was only 0.1–0.3% in 861 yak (81.92%), likely corresponding to background levels (Kaeuffer *et al.* 2007). It, however, reached a value of 32.9% in an individual of the North Hangai population. A total of 14 yak individuals showed values ranging from 12.5% to 32.9% of inferred cattle admixture, and 91 individuals had values ranging from 1.00% to 9.80% of inferred cattle admixture.

### Relationship between the level of cattle introgression and altitude

Yak-cattle hybrid F_1_ animals are most popular in agro-pastoral areas at altitudes ranging from 1500 to 2500 metres (Zhang 1989), and therefore a hybridization zone might be expected at and around these altitudes. Although admixture estimates of *mY* and *Q*_cattle_ showed a clear geographical structure, with higher levels of admixture in the QTP and M&R regions and lower levels in the HPP region (Fig. 1c and d), our results indicated that there was no significant correlation (*P*>0.05) between the level of cattle admixture in terms of mtDNA or microsatellite diagnostic markers, *mY* or *Q*_cattle_ estimates and the altitude across geographical regions (Fig. S2) and within geographical region (Fig. S3).

## Discussion

Ancient nomadic people are believed to have started hybridization of yak with cattle 3000 years ago (Cai 1989; Zhang 1989, 2000). Cattle bulls are commonly used to hybridize with yak cows at relatively high altitudes, while reciprocal crossing is practiced at low altitudes of their distribution range (e.g. Phillips *et al.* 1946a,b; Cai 1980; Joshi 1982; Zhang 1989; Adachi & Kawamoto 1992; Davaa 1996; Tshering *et al.* 1996). Hybrid males are sterile and their fertility does not resume until the fourth backcrossing generation (Deakin *et al.* 1935; Cai 1989; Tumennasan *et al.* 1997; Zhang 2000; Hisabumi *et al.* 2002), and therefore male-mediated cattle introgression in yak is impossible (e.g. Jianlin *et al.* 2002) and thus the cattle genes are only introduced into yak genome by hybridization of female F_1_ hybrids to yak. Consequently, *Y*-chromosome–specific markers are not helpful in detecting cattle introgression in domestic yak.

In this study, we first chose a cattle-specific mtDNA control region fragment (Ward *et al.* 1999) and cattle-specific alleles at three autosomal microsatellite loci (*ILSTS013*, *ILSTS050* and *SPS115*) for a diagnostic approach to assess the impact of cattle introgression on domestic yak populations. We also chose an estimator of admixture proportion and a model-based Bayesian admixture analysis, two methods that have been widely used for estimating the admixture proportion between closely related species (Hanotte *et al.* 2002; Freeman *et al.* 2004; Edwards *et al.* 2007), in order to estimate cattle admixture proportion in the yak genetic pool at the genome level. The *mY* estimator was chosen as it appears suitable for estimating admixture proportion using molecular data. The *mY* has also the advantages of no bias and relatively low variance, in comparison to two other conventional estimators of *mR* (Roberts & Hiorns 1965) and *mC* (Chakraborty *et al.* 1992), which only consider gene frequencies. Bayesian admixture analysis requires no prior information on the identity of possible parental populations or on the possible assignment of individuals into populations. It was performed using allelic information at 17 microsatellite loci.

The diagnostic marker-based approach detected cattle introgression in 22 out of 29 yak populations with an average frequency of 11.8%, and 127 individuals showed cattle-specific mtDNA sequences and/or autosomal microsatellite alleles. The QTP group had a significantly higher frequency of cattle introgression than the M&R group, followed by the HPP group (*P* < 0.01). The frequency of cattle introgression in the surrounding QTP subgroup was significantly higher than that in the heartland QTP subgroup (*P* < 0.05). Although the diagnostic marker approach identified a relatively high incidence of cattle introgression in contemporary domestic yak populations, it does not allow us to estimate the level of cattle introgression into the yak genome. We therefore applied two admixture analyses using allelic information at 17 microsatellite loci dispersed throughout the genome. Both *mY* admixture and Bayesian admixture estimations indicated that the average proportion of cattle admixture in the contemporary domestic yak genome was generally low at a population level. However, it varied a lot among the populations and geographical groups, and also among individuals within a population.

Out of the 17 yak detected with taurine mtDNA sequences, only two also had a cattle allele at one of the three diagnostic microsatellites loci and only three showed a *Q*_cattle_ above 0.3% (Table S2). It is therefore important to combine information from genetic markers with different modes of inheritances as well as to perform data analyses using different statistical approaches in order to assess introgression between yak and cattle. Interestingly, while the large variation of cattle admixture across individuals within population suggests an ongoing process of cattle introgression under the assumption that genetic imprints of ancient cattle introgression would be ‘homogenized’ at the yak genome level, the detection of yak individuals with only cattle mtDNA sequences is also an indication of ancient introgression events.

Yak pastoralism is a transhumant and seasonal activity, and yak herders usually keep yak-cattle F_1_ hybrid and backcross animals as packing, riding or draught animals (Wiener *et al.* 2003). Traditionally, hybridization between yak and cattle and backcrossing of F_1_ hybrid females are driven to produce the F_1_ and first generation (B_1_) of backcross hybrids only (Zhang 1989, 2000; Wiener *et al.* 2003), and therefore a high frequency of cattle introgression in domestic yak populations is not expected. We observed, however, a high incidence of cattle introgression in contemporary domestic yak populations, typically in the QTP and M&R groups where F_1_ and first generation of backcross (B_1_) hybridization have been practiced for thousands of years (Zhang 1989, 2000; Davaa 1996; Wiener *et al.* 2003). This suggests that these practices had and still have an impact on the yak genetic integrity. Unattended management of free-ranging animals, especially during the breeding seasons in summer pastures, may account for the high frequency of cattle introgression in yak populations (Phillips *et al.* 1946a,b; Wiener *et al.* 2003).

In addition, other factors such as the geographical locations where yak pastoralism is practiced, founder effect and breeding strategy may explain the variations of frequency of cattle introgression among yak populations within and among geographical areas. For example, yak-cattle hybridization is not common in the pastoral areas at high elevations where cattle cannot adapt well, while it is widespread in areas of agro-pastoral zone at relatively low altitudes (Wiener *et al.* 2003). This is consistent with our observations that the surrounding QTP subgroup displayed a significantly higher frequency of cattle introgression than the heartland QTP subgroup. In particular, the highest frequency of cattle introgression was detected in the Jiulong (37.50%) and Maiwa (41.67%) populations, which are located in the surrounding QTP areas. In the case of the Maiwa yak, hybridization between yak and taurine cattle has been widely carried out to improve its milk production (Cai 1980, 1989), while Jiulong yak are the descendants of a small population that survived a severe outbreak of rinderpest 150 years ago (Wiener *et al.* 2003). A contemporary population of 50 000 yak was developed from a small population of survivors, and hybridization between yak and local cattle may have occurred in the process of population recovery and expansion, with a closed breeding programme being responsible for the high frequency of individuals carrying cattle-specific mtDNA sequences and/or autosomal microsatellite alleles in the population.

A generally low frequency of cattle introgression was observed in the HPP group distributed at relatively high elevations (approximately 3500 metres), where yak-cattle hybrids seem to be unattractive to the pastoralists because of their poor adaptability to this habitat (Pal & Madan 1996; Rasool *et al.* 2002). This confirms the claim that hybridization is rare in the majority of HPP yak populations except for the Indian, Bhutanese and Nepalese Himalayan areas where hybridization between yak and taurine or indicine cattle has been reported (Joshi 1982; Pal & Madan 1996; Sherchand & Karki 1996; Dorji *et al.* 2002; Wiener *et al.* 2003).

Although hybridization is supposedly being practiced with local cattle populations in wide yak-rearing areas, the European taurine cattle have also been used for the exercise since 1940s (Wiener *et al.* 2003). Our mtDNA-based approach indicated that all cattle mtDNA sequences detected in yak populations were of T3 taurine cattle (Troy *et al.* 2001), which are equally predominant in both European and Chinese cattle populations, and therefore the respective impact of European or local cattle introgression on domestic yak populations cannot be assessed. Also, it should be noted that while we failed to sequence the mtDNA of two Maiwa yaks showing a diagnostic cattle fragment, the absence of any indicine cattle within or around the current distribution of Maiwa yak does make it unlikely that there is any zebu cattle introgression in this population. Our study clearly illustrates the impact of taurine cattle introgression into domestic yak, although it does not provide any evidence of zebu introgression into yak populations. It is well-known that yak-cattle hybridization is primarily driven to produce F_1_ and B_1_ hybrids, and our results indicate the presence of gene flow between yak and cattle in the majority of contemporary yak populations. Our findings suggest that cattle introgression is an ongoing process and might have been relatively more important in recent times. To protect yak genetic integrity, the hybridization between yak and cattle should therefore be tightly controlled.

## Supporting information

**Figure S1** Allele spectra of 17 microsatellite loci in yak, cattle and their F_1_ hybrid populations. The *Y*-axis represents the allele frequency and the *X*-axis the allele sizes.

**Figure S2** Correlation between the level of cattle admixture and the altitude of geographic location across geographic regions.

**Figure S3** Correlation between the level of cattle admixture and the altitude of geographic location within geographic region.

**Table S1** Seventeen microsatellite loci and their PCR conditions and allele size ranges in domestic yak populations.

**Table S2** Yak individuals showing cattle-specific mtDNA and microsatellite alleles and *Q*_cattle_ proportions.

**Appendix S1** Supplementary methods and results.

As a service to our authors and readers, this journal provides supporting information supplied by the authors. Such materials are peer-reviewed and may be re-organized for online delivery, but are not copy-edited or typeset. Technical support issues arising from supporting information (other than missing files) should be addressed to the authors.

## References

[b1] Adachi A, Kawamoto Y (1992). Hybridization of yak and cattle among the Sherpas in Solu and Khumbu, Nepal. Report of the Society for Researches on Native Livestock.

[b2] Bertorelle G, Excoffier L (1998). Inferring admixture proportions from molecular data. Molecular Biology and Evolution.

[b3] Cai L (1980). The nomenclature for the hybrids of yak with cattle (in Chinese). Journal of China Yak.

[b4] Cai L (1989). Sichuan Yak.

[b5] Chakraborty R, Kamboh MI, Nwankwo M, Ferrell RE (1992). Caucasian genes in American blacks: new data. American Journal of Human Genetics.

[b6] Davaa M (1996). Conservation and Management of Domestic yak Genetic Diversity in Mongolia.

[b7] Deakin A, Muir GW, Smith AG (1935). Hybridisation of domestic cattle, bison and yak. Report of the Wainwright Experiment, Department of Agricuture, Dominion of Canada.

[b8] Derr JN, Davis SK, Woolley JB, Wharton RA (1992). Variation and the phylogenetic utility of the large ribosomal subunit of mitochondrial DNA from the insect order Hymenoptera. Molecular Phylogenetics and Evolution.

[b9] Dorji T, Goddard M, Perkins J, Robinson N, Roder W (2002). Genetic Diversity in Bhutanese yak (Bos Grunniens) Populations Using Microsatellite Markers.

[b10] Dupanloup I, Bertorelle G (2001). Inferring admixture proportions from molecular data: extension to any number of parental populations. Molecular Biology and Evolution.

[b11] Edwards CJ, Baird JF, MacHugh DE (2007). Taurine and zebu admixture in Near Eastern cattle: a comparison of mitochondrial, autosomal and Y-chromosomal data. Animal Genetics.

[b12] Feng DM, Zhao HJ, Jianlin H (2009). Species specific alleles at three microsatellite loci in yak and cattle. Acta Veterinaria et Zootechnica Sinica.

[b13] Freeman AR, Meghen CM, MacHugh DE, Loftus RT, Achukwi MD, Bado A, Sauveroche B, Bradley DG (2004). Admixture and diversity in West African cattle populations. Molecular Ecology.

[b14] Hanotte O, Bradley DG, Ochieng JW, Verjee Y, Hill EW, Rege JEO (2002). African pastoralism: genetic imprints of origins and migrations. Science.

[b15] Hisabumi T, Tumennasan K, Kazuyuki H, Chandley AC, Yasuo H (2002). Fertility investigation in F_1_ hybrid and backcross progeny of cattle (*Bos taurus*) and yak (*B. gruniens*) in Mongolia.: II. Little variation in gene products studied in male sterile and fertile animals. Niigata Journal of Health and Welfare.

[b16] Jianlin H, Ochieng JW, Rege JEO, Hanotte O (2002). Low Level of Cattle Introgression in yak Populations From Bhutan and China: Evidences From Y-Specific Microsatellites and Mitochondrial DNA Markers.

[b17] Joshi DD (1982). Yak and Chauri Husbandry in Nepal.

[b18] Kaeuffer R, Reale D, Coltman DW, Pontier D (2007). Detecting population structure using STRUCTURE software: effect of background linkage disequilibrium. Heredity.

[b19] Lai SJ, Liu YP, Liu YX, Li XW, Yao YG (2006). Genetic diversity and origin of Chinese cattle revealed by mtDNA D-loop sequence variation. Molecular Phylogenetics and Evolution.

[b20] Lai SJ, Chen SY, Liu YP, Yao YG (2007). Mitochondrial DNA sequence diversity and origin of Chinese domestic yak. Animal Genetics.

[b21] Nguyen TT, Genini S, Menetrey F, Malek M, Vogeli P, Goe MR, Stranzinger G (2005). Application of bovine microsatellite markers for genetic diversity analysis of Swiss yak (*Poephagus grunniens*). Animal Genetics.

[b22] Pal RN, Madan ML (1996). Yak Production in India.

[b23] Phillips RW, Tolstoy IA, Johnson RG (1946a). Yaks and yak-cattle hybrids in Asia. Journal of Heredity.

[b24] Phillips RW, Tolstoy IA, Johnson RG (1946b). Yaks and yak-cattle hybrids in Asia. Journal of Heredity.

[b25] Pritchard JK, Stephens M, Donnelly P (2000). Inference of population structure using multilocus genotype data. Genetics.

[b26] Qi X-B (2004). Genetic Diversity, Differentiation and Relationship of Domestic yak Populations - a Microsatellite and Mitochondrial DNA Study.

[b27] Rasool G, Khan BA, Jasra AW (2002). Yak Pastoralism in Pakistan.

[b28] Ritz LR, Glowatzki-Mullis ML, MacHugh DE, Gaillard C (2000). Phylogenetic analysis of the tribe Bovini using microsatellites. Animal Genetics.

[b29] Roberts DF, Hiorns RW (1965). Methods of analysis of the genetic composition of a hybrid population. Human Biology.

[b30] Sambrook J, Fritsch EF, Maniatis T (1989). Molecular Cloning: A Laboratory Manual.

[b31] Sherchand L, Karki NPS (1996). Conservation and Management of yak Genetic Diversity in Nepal.

[b32] The Editing-Committee (1989). China Yakology.

[b33] Troy CS, MacHugh DE, Bailey JF, Magee DA, Loftus RT, Cunningham P, Chamberlain AT, Sykes BC, Bradley DG (2001). Genetic evidence for Near-Eastern origins of European cattle. Nature.

[b34] Tshering L, Gyamtsho P, Gyeltshen T (1996). Yaks in Bhutan.

[b35] Tumennasan K, Tuya T, Hotta Y, Takase H, Speed RM, Chandley AC (1997). Fertility investigations in the F_1_ hybrid and backcross progeny of cattle (*Bos taurus*) and yak (*B. grunniens*) in Mongolia. Cytogenetics and Cell Genetics.

[b36] Ward TJ, Bielawski JP, Davis SK, Templeton JW, Derr JN (1999). Identification of domestic cattle hybrids in wild cattle and bison species: a general approach using mtDNA markers and the parametric bootstrap. Animal Conservation.

[b37] White WT, Phillips RW, Elting EC (1946). Yaks and yak-cattle hybrids in Alaska. Journal of Heredity.

[b38] Wiener G, Jianlin H, Ruijun L (2003). The Yak. The Regional Office for Asia and the Pacific.

[b39] Xuebin Q, Jianlin H, Rege JEO, Hanotte O (2002). Y-Chromosome Specific Microsatellite Polymorphisms in Chinese yak.

[b40] Xuebin Q, Jianlin H, Lkhagva B, Chekarova I, Badamdorj D, Rege JE, Hanotte O (2005). Genetic diversity and differentiation of Mongolian and Russian yak populations. Journal of Animal Breeding and Genetics.

[b41] Zhang RC (1989). China Yak.

[b42] Zhang RC, Zhao XX, Zhang RC (2000). Interspecies hybridization between yak, *Bos taurus* and *Bos indicus* and reproduction of the hybrids. Recent advances in yak reproduction.

