# Supplementary material for: Assessment of cattle genetic introgression into domestic yak populations using mitochondrial and microsatellite DNA markers
Source: Anim Genet. 2010 Jun;41(3):242–52. doi: 10.1111/j.1365-2052.2009.01989.x (PMC2878598; doi:10.1111/j.1365-2052.2009.01989.x)
Supplement: Supplementary file 1 [file age0041-0242-SD1.pdf]

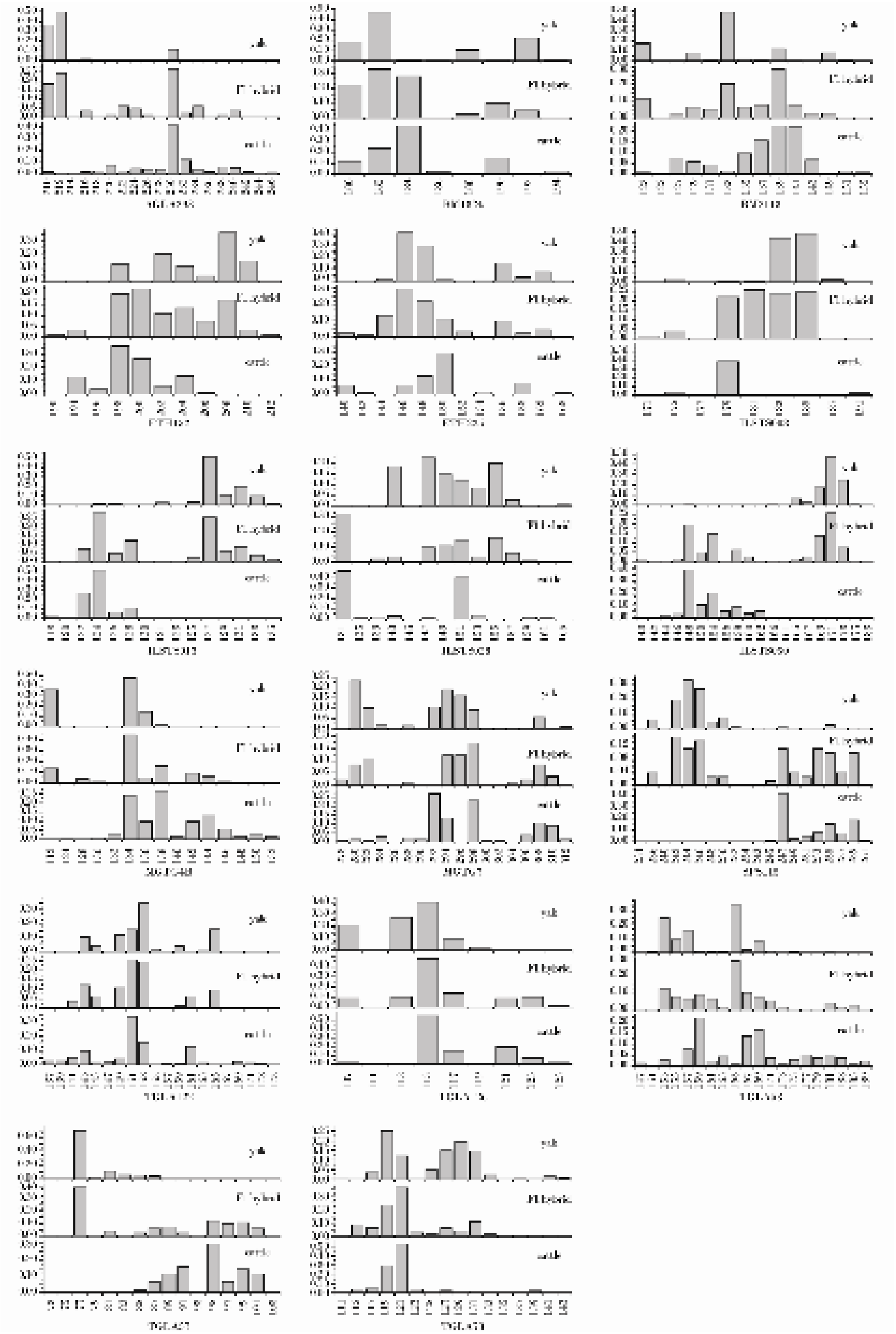

Figure S1. Allele spectra of 17 microsatellite loci in yak, cattle and their F_1 hybrid populations. The *Y*-axis represents the allele frequency and the *X*-axis the allele sizes.
