# Supplementary material for: Assessment of cattle genetic introgression into domestic yak populations using mitochondrial and microsatellite DNA markers
Source: Anim Genet. 2010 Jun;41(3):242–52. doi: 10.1111/j.1365-2052.2009.01989.x (PMC2878598; doi:10.1111/j.1365-2052.2009.01989.x)
Supplement: Supplementary file 2 [file age0041-0242-SD2.pdf]

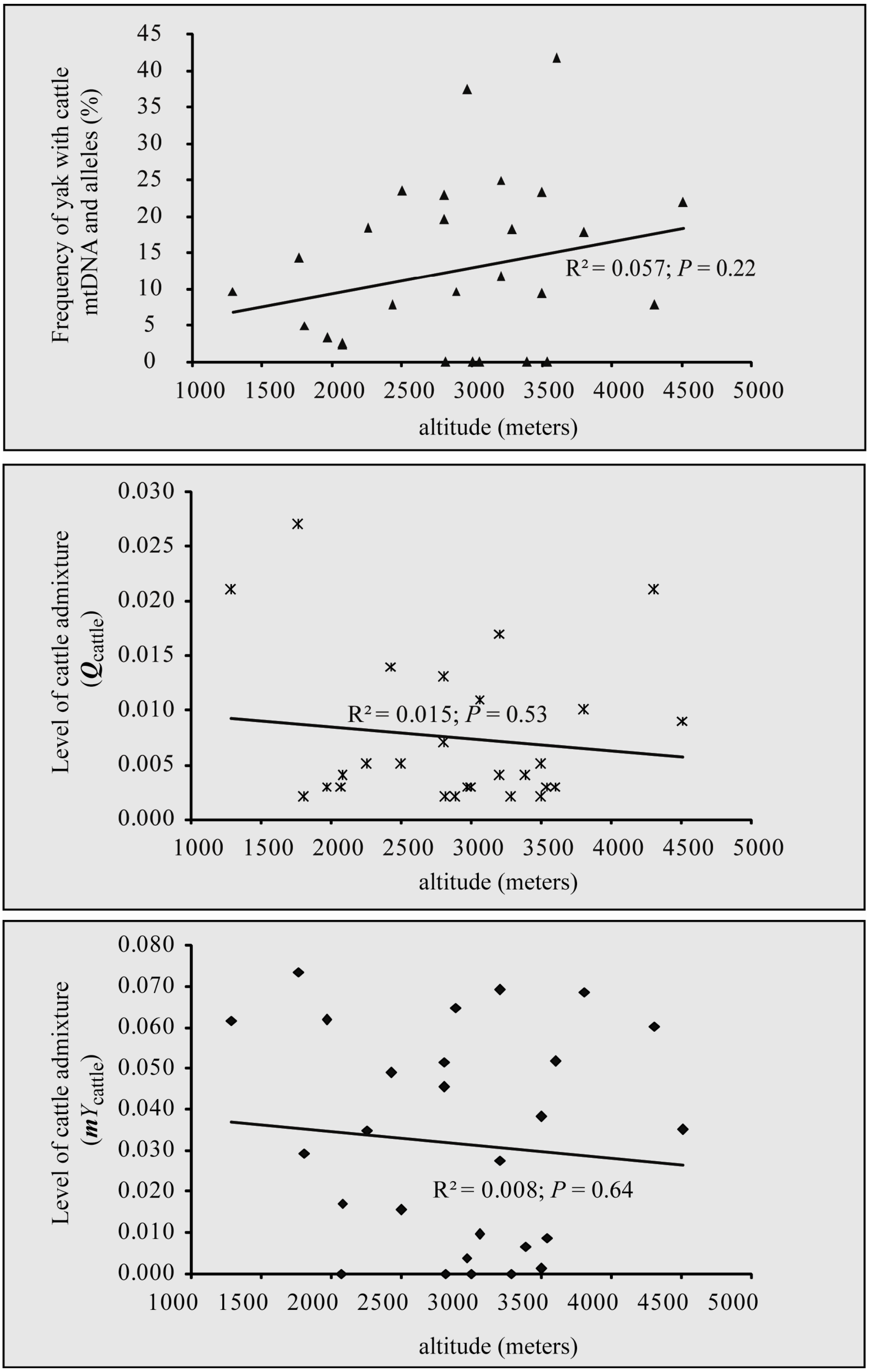

Figure S2. Correlation between the level of cattle admixture and the altitude of geographic location across geographic regions.
