# Supplementary material for: Assessment of cattle genetic introgression into domestic yak populations using mitochondrial and microsatellite DNA markers
Source: Anim Genet. 2010 Jun;41(3):242–52. doi: 10.1111/j.1365-2052.2009.01989.x (PMC2878598; doi:10.1111/j.1365-2052.2009.01989.x)
Supplement: Supplementary file 4 [file age0041-0242-SD4.pdf]

Table S1. Seventeen microsatellite loci and their PCR conditions and allele size ranges in domestic yak populations

Locus Name	Chr*	Primer sequence (5' - 3')<br>Forward (above) and Reverse (below)	A.T. (°C)	Forward primer Label	Allele range (base pairs)
AGLA293<br>(D5S13)	5	GAAACTCAACCCAAGACAACTCAAG<br>ATGACTTTATTCTCCACCTAGCAGA	55	FAM	210-240
BM1824<br>(D1S34)	1	GAGCAAGGTGTTTTTCCAATC<br>CATTCTCCAACTGCTTCCTTG	58	TET	180-194
BM2113<br>(D2S26)	2	GCTGCCTTCTACCAAATACCC<br>CTTCCTGAGAGAAGCAACACC	58	TET	123-151
ETH152<br>(D5S1)	5	TACTCGTAGGGCAGGCTGCCTG<br>GAGACCTCAGGGTTGGTGATCAG	55	TET	194-212
ETH225<br>(D9S1)	9	GATCACCTTGCCACTATTTCCT<br>ACATGACAGCCAGCTGCTACT	65	FAM	144-162
ILSTS008<br>(D14S15)	14	GAATCATGGATTTTCTGGGG<br>TAGCAGTGAGTGAGGTTGGC	58	FAM	175-187
ILSTS013<br>(D9S10)	9	CTTGATCCTTATAGAACTGG<br>ACACAAAATCAGATCAGTGG	58	HEX	121-135
ILSTS028<br>(D11S63)	11	TCCAGATTTTGTACCAGACC<br>GTCATGTCATACCTTTGAGC	55	FAM	131-165
ILSTS050<br>(D2S45)	2	AAATCAGACACCCAGTTTCC<br>GTTTTTCTACACGAGTTGGC	55	TET	161-183
MGTG4B<br>(D4S5)	4	GAGCAGCTTCTTTCTTTCTCATCTT<br>GCTCTTGGAAGCTTATTGTATAAAG	55	FAM	112-138
MGTG7<br>(D23S5)	23	TTCATTGCAGCACTATTTACAATAG<br>TAAGTTCCCTGTATCATTTTTTGAA	55	TET	278-312
SPS115	15	AAAGTGACACAACAGCTTCTCCAG<br>AACGAGTGTCCTAGTTTGGCTGTG	65	TET	234-254
TGLA122<br>(D21S6)	21	CCCTCCTCCAGGTAAATCAGC<br>AATCACATGGCAAATAAGTACATAC	55	HEX	143-175
TGLA126<br>(D20S1)	20	CTAATTTAGAATGAGAGAGGCTTCT<br>TTGGTCTCTATTCTCTGAATATTCC	55	HEX	107-121
TGLA53<br>D16S3)	16	GCTTTCAGAAATAGTTTGCATTCA<br>ATCTTCACATGATATTACAGCAGA	55	TET	151-181
TGLA57<br>(D1S8)	1	GCTTTTTAATCCTCAGCTTGCTG<br>GCTTCCAAAACTTTACAATATGTAT	55	FAM	77-99
TGLA73<br>(D9S3)	9	GAGAATCACCTAGAGAGAGGCA<br>CTTTCTCTTTAAATTCTATATGGT	55	FAM	111-143

* Cattle chromosome assignments are given.

A.T., annealing temperature for PCR amplification.
