# Supplementary material for: Assessment of cattle genetic introgression into domestic yak populations using mitochondrial and microsatellite DNA markers
Source: Anim Genet. 2010 Jun;41(3):242–52. doi: 10.1111/j.1365-2052.2009.01989.x (PMC2878598; doi:10.1111/j.1365-2052.2009.01989.x)
Supplement: Supplementary file 5 [file age0041-0242-SD5.pdf]

Table S2. Yak individuals showing cattle-specific mtDNA and microsatellite alleles and Q_{cattle} proportions

yak individuals	Qcattle	Qyak	mtDNA	ILSTS013	ILSTS050	SPS115
AH12	0.045	0.955				Yes
AH28	0.329	0.671		Yes	Yes	
AH30	0.039	0.961			Yes	
AH32	0.145	0.855	Yes			
AH46	0.238	0.762		Yes	Yes	
AH55	0.002	0.998	Yes			
AH58	0.294	0.706		Yes		
AS07	0.002	0.998		Yes		
AS27	0.002	0.998		Yes		
AS33	0.017	0.983			Yes	
BZ04	0.026	0.974				Yes
BZ19	0.002	0.998				Yes
BZ34	0.019	0.981				Yes
BZ42	0.002	0.998				Yes
CB03	0.001	0.999	Yes			
CB04	0.161	0.839				Yes
CB28	0.04	0.96		Yes		
DT09	0.002	0.998				Yes
DT13	0.001	0.999				Yes
DT17	0.014	0.986			Yes	
DT25	0.003	0.997				Yes
DT26	0.047	0.953			Yes	
DT32	0.002	0.998	Yes			
DT36	0.026	0.974	Yes	Yes		
GA02	0.181	0.819		Yes		
GA11	0.244	0.756		Yes		
GA26	0.003	0.997	Yes			
HG13	0.001	0.999		Yes, homozygote		
JZ03	0.063	0.937				Yes
JZ04	0.025	0.975				Yes
JZ05	0.002	0.998				Yes
JZ09	0.024	0.976				Yes
JZ15	0.004	0.996				Yes
JZ16	0.006	0.994				Yes
JZ32	0.003	0.997				Yes
JZ35	0.009	0.991		Yes		Yes
KG16	0.016	0.984				Yes
LQ10	0.009	0.991				Yes
LQ13	0.002	0.998				Yes
LQ14	0.002	0.998	Yes			
LQ18	0.007	0.993				Yes
LQ27	0.002	0.998				Yes
LQ28	0.002	0.998				Yes
LQ32	0.003	0.997				Yes
MQ08	0.002	0.998				Yes
MQ11	0.002	0.998				Yes
MQ14	0.002	0.998				Yes
MQ15	0.125	0.875			Yes	
MQ27	0.021	0.979		Yes		

MQ31	0.018	0.982				Yes
MQ34	0.005	0.995				Yes
MQ45	0.027	0.973				Yes, Homozygote
MW05	0.011	0.989		Yes		
MW06	0.012	0.988		Yes		
MW17	0.016	0.984		Yes		
MW20	0.002	0.998				Yes
MW21	0.003	0.997				Yes
MW23	0.098	0.902		Yes, homozygote		
MW27	0.003	0.997				Yes
MW30	0.002	0.998				Yes
MW32	-	-	Yes	-	-	-
MW39	-	-	Yes	-	-	-
NI01	0.005	0.995		Yes		
NI03	0.002	0.998		Yes		
NI07	0.016	0.984		Yes		
NI08	0.007	0.993		Yes		
NI09	0.008	0.992		Yes		
NI15	0.002	0.998		Yes		
NI19	0.004	0.996		Yes		
NI50	0.033	0.967				Yes
RU09	0.002	0.998	Yes			
RU17	0.013	0.987		Yes, homozygote		
SJ07	0.048	0.952		Yes		
SJ08	0.146	0.854		Yes, homozygote		
SJ11	0.146	0.854		Yes		
SJ12	0.002	0.998	Yes			
SJ15	0.002	0.998				Yes
SJ18	0.009	0.991		Yes		
SJ21	0.01	0.99		Yes		
SJ22	0.07	0.93		Yes, homozygote		
SJ26	0.042	0.958		Yes		
SN03	0.002	0.998				Yes
SN11	0.011	0.989	Yes			
SN12	0.008	0.992		Yes		
SN13	0.002	0.998	Yes			
SN15	0.011	0.989				Yes
SN19	0.015	0.985				Yes
SN22	0.003	0.997				Yes
SN29	0.007	0.993		Yes		
SN38	0.003	0.997				Yes, Homozygote
TB02	0.001	0.999	Yes			
TB05	0.002	0.998	Yes	Yes		
TB13	0.002	0.998	Yes			
TB18	0.003	0.997	Yes			
TB25	0.007	0.993				Yes
TB31	0.048	0.952			Yes	

TB36	0.002	0.998	Yes				
TB45	0.006	0.994		Yes			
TB47	0.011	0.989				Yes	
TJ05	0.005	0.995		Yes		Yes	
TJ06	0.006	0.994				Yes	
TJ09	0.014	0.986				Yes	
TJ16	0.002	0.998				Yes	
TJ20	0.01	0.99				Yes	
TJ22	0.049	0.951				Yes	
TJ27	0.004	0.996				Yes	
TJ31	0.002	0.998				Yes	
TJ39	0.002	0.998				Yes	
TJ42	0.002	0.998				Yes	
TJ47	0.002	0.998				Yes	
TW13	0.002	0.998				Yes	
TW17	0.002	0.998				Yes	
TW18	0.002	0.998				Yes	
TW25	0.006	0.994				Yes	
TW26	0.002	0.998				Yes	
TW27	0.002	0.998				Yes	
TW36	0.001	0.999			Yes		
TW39	0.004	0.996			Yes		
TW40	0.003	0.997			Yes		
TW44	0.002	0.998				Yes	
TW50	0.002	0.998	Yes				
UB07	0.04	0.96			Yes		
UG19	0.309	0.691				Yes	
UG26	0.042	0.958		Yes			
UG28	0.204	0.796		Yes			
XH08	0.076	0.924				Yes	
XH16	0.002	0.998				Yes	

Note: -, No data available.
