# Supplementary material for: Assessment of cattle genetic introgression into domestic yak populations using mitochondrial and microsatellite DNA markers
Source: Anim Genet. 2010 Jun;41(3):242–52. doi: 10.1111/j.1365-2052.2009.01989.x (PMC2878598; doi:10.1111/j.1365-2052.2009.01989.x)
Supplement: Supplementary file 6 [file age0041-0242-SD6.pdf]

Appendix S1. Supplementary methods and results.

Mitochondrial DNA control region amplification and sequencing

Cattle mitochondrial DNA (mtDNA) in yak was detected through the amplification of a 357-bp cattle (*B. taurus* and *B. indicus*)-specific mtDNA control region fragment, with primers MTD1 (5'-AGCTAACATAACACGCCCATAC-3') and MTD2 (5'-CCTGAAGAAAGAACCAGATGC-3') (Ward *et al.* 1999) in a multiplex PCR reaction with primers MTR1 (5'-CCCGCCTGTTTATCAAAAACAT-3') and MTR2 (5'-CCCTCCGGTTTGAACTCAGAT-3') (Derr *et al.* 1992), which amplified a 590-bp mammalian conserved 16S rDNA fragment, as internal control.

PCR reactions were carried out in 10 μl volume comprising 20-50 ng of genomic DNA, 5 pmol of each primer, 0.5 units of *Taq* DNA polymerase (Promega, Wisconsin, USA), 0.15 mM of each dNTPs and 1 x PCR buffer containing 10 mM Tris-HCl (pH 8.3), 50 mM KCl, 0.001% gelatin (Sigma), 0.25% Nonidet P40 (BDH) and 2.0 mM $MgCl_2$. PCR amplifications were performed on a GeneAmp PCR System 9700 thermocycler (Applied Biosystems, California, USA) as follows: an initial denaturation step at 95°C for 5 min followed by 35 cycles of 95°C for 30 s, 55°C for 1 min and 72°C for 1 min and a final extension step at 72°C for 7 min. PCR products were separated on 2% (w/v) agarose gel containing ethidium bromide (10 ng/ml) for 2 hrs at 80 V in a 1 x TBE buffer.

To further clarify the origin taurine or zebu of cattle mtDNAs detected in domestic yak populations, a control region fragment of these mtDNAs was amplified using the published forward primer proline tRNA (5'-CTGCAGTCTCACCATCAACC-3') (Loftus *et al.* 1994) and reverse primer MTD2 (Ward *et al.* 1999) in 50 μl volume under the same PCR conditions described above. PCR products were purified using QIAquick PCR purification column (QIAGEN, Germany) according to the manufacturer's instructions. Direct sequencing of both strands of 486-bp PCR products were performed with the same PCR primers using the ABI PRISM Dye Terminator Cycle Sequencing Ready Reaction Kit (Applied Biosystems, California, USA) in 20 μl volume comprising approximately 70 ng of purified PCR product and 3.2 pmol of primer. Sequencing reactions were carried out in 25 cycles of 10 s at 96°C, 5 s at 50°C, and 4 min at 60°C on the GeneAmp PCR System 9700 thermocycler. Reaction products were separated on an ABI 377 PRISM DNA sequencer (Applied Biosystems, California, USA) in a 5% Long Ranger Gel (FMC). The sequences of novel haplotypes have been deposited in the GenBank with accession numbers AY428633-AY428643.

Analysis of cattle-specific microsatellite alleles in domestic yak populations

As illustrated in Figure S1, the allelic genotype patterns overlapped between yak and cattle for 14 out of 17 microsatellite loci genotyped, and it was not possible to determine the cattle-specific alleles at these loci. However, three microsatellite loci (*ILSTS013*, *ILSTS050* and *SPS115*) yielded allele sizes that were completely distinct between yak and cattle. More particularly, allele sizes at *ILSTS013* and *ILSTS050* were even numerals ranging from 118 bp to 130 bp and from 140 bp to 166 bp in cattle, respectively, but odd numerals ranging from 121 bp to 135 bp and 161 bp to 183 bp were observed in yak, respectively. At *SPS115*, allele sizes were odd numerals ranging from 243 bp to 261 bp in cattle but even numerals ranging from 234 bp to 254 bp in yak. These alleles have been further sequenced to confirm their size differences between yak and cattle (Feng *et al*. 2009).

References

Derr J.N., Davis S.K., Woolley J.B. & Wharton R.A. (1992) Variation and the phylogenetic utility of the large ribosomal subunit of mitochondrial DNA from the insect order Hymenoptera. *Mol. Phylogenet. Evol.* **1**, 136-47.

Feng D.M., J. Z.H. & Jianlin H. (2009) Species specific alleles at three microsatellite loci in yak and cattle. *Veterianria et Zootechnica Sinica*, in press.

Loftus R.T., MacHugh D.E., Bradley D.G., Sharp P.M. & Cunningham P. (1994) Evidence for two independent domestications of cattle. *Proc. Natl. Acad. Sci. USA* **91**, 2757-61.

Ward T.J., Bielawski J.P., Davis S.K., Templeton J.W. & Derr J.N. (1999) Identification of domestic cattle hybrids in wild cattle and bison species: a general approach using mtDNA markers and the parametric bootstrap. *Animal Conservation* **2**, 51-7.
